# AMBRA1 attenuates the proliferation of uveal melanoma cells

**DOI:** 10.1515/med-2021-0386

**Published:** 2021-11-27

**Authors:** Binbin Zhao, Yun Yang, Biyun Cun, Ping Chen

**Affiliations:** Department of Ophthalmology, Renji Hospital, School of Medicine, Shanghai Jiaotong University, Shanghai, 200127, China; Center for Excellence in Molecular Cell Science, Chinese Academy of Sciences, Shanghai, 200031, China; Department of Clinical Skills Center, Kunming Medical University, Kunming, 650500, Yunnan, China

**Keywords:** uveal melanoma, AMBRA1, CCND1, ubiquitination

## Abstract

Uveal melanoma (UVM) is the most common primary intraocular malignancy in adults with high metastasis rates. D-type cyclins (CCNDs) are central regulators of the cell division cycle and are among the most frequently deregulated therapeutic targets in human cancer. Recently, the E3 ligase adaptor, autophagy and beclin 1 regulator 1 (AMBRA1), was reported to regulate the stability of CCNDs, including CCND1, but its role in UVM has not been demonstrated. AMBRA1 is lowly expressed in UVM cells, and the ablation of AMBRA1 promotes the proliferation of 92.1 and OMM1 cells, whereas ectopically expressing AMBRA1 attenuates the proliferation of UVM cells. Further studies found that AMBRA1 promotes the ubiquitination and degradation of CCND1, and AMBRA1 regulates the proliferation of UVM cells in a CCND1-dependent manner. Thus, this study suggests that AMBRA1 serves as an important tumor suppressor by limiting UVM cell growth.

## 1 Introduction

Uveal melanoma (UVM) is a melanoma of the eyes, which arises from melanocytes in the uvea, 90% of which involve the choroid, 6% of which are confined to the ciliary body, and 4% of which involve the iris [1,2]. Approximately, 50% of patients diagnosed with UVM will develop metastasis within 10 years from the primary intraocular tumor [3,4]. The majority of UVM is thought to occur as sporadic [5], meaning everyone is at risk of developing UVM. The causes of somatic mutations in UVM, including sun exposure, radiation, cigarette smoking, and certain chemicals, are known to increase the risk of mutations in genes that control cancers [1].

The ocular treatment usually aims to conserve the eyeball, preserving the visual performance. The treatment of primary UVM includes phototherapy, radiotherapy, and local resection and reservation of enucleation in advanced cases [1]. So far, no standard treatment exists to reduce the risk of metastasis and improve the overall survival of patients [6]. Targeted therapy is widely used in cancer treatment due to its lesser side effects compared to other strategies. Novel therapeutics targeting mutant guanine nucleotide-binding protein G(q) subunit alpha/guanine nucleotide-binding protein subunit alpha-11, regulators of G-protein signaling, inactivated BAP1, and immune-checkpoint blockade is emerging [1]. Different inhibitors specifically target particular mechanisms of UVM occurrence and progression. Thus, new targets for melanoma treatment are urgently needed.

Protein ubiquitination is a dynamic multifaceted posttranslational modification involved in nearly all aspects of eukaryotic biology, including cancer development and progression, cell survival and differentiation, innate and adaptive immunity, and individual development and sexual maturity [7,8,9]. Ubiquitination includes a sequence of three enzymatic steps, and aberrations in the pathway can lead to tumor development and progression as observed in many cancer types [10], including UVM. BAP1 is a deubiquitinating enzyme, and its somatic and germline mutations have been found in UVM, malignant mesothelioma, and clear-cell renal cell carcinoma tumors [11].

D-type cyclins are central regulators of the cell division cycle and are among the most frequently deregulated therapeutic targets in human cancer [12]. The autophagy and beclin 1 regulator 1 (AMBRA1) was identified as the main regulator of cyclin D in several earlier studies, and it mediates ubiquitination and degradation of cyclin D as a substrate receptor for the cullin 4 E3 ligase complex [13,14]. The role of AMBRA1 in UVM has not been reported. In this study, the function of AMBRA1 in UVM was extensively studied.

## 2 Materials and methods

### 2.1 Cell culture and transfection

The normal human pigment epithelial cell line ARPE-19 and three human UVM cell lines 92.1, MuM2B, and OMM1 were kindly gifted from Professor Shengfang Ge (Shanghai Jiao Tong University, China). All these cells were cultured in Dulbecco's Modified Eagle Medium (Hyclone, USA), supplemented with 10% fetal bovine serum (Gibco, USA) and 100 U/mL penicillin and 100 mg/mL streptomycin (Gibco), in a 37°C humidified atmosphere of 5% CO_2_. Plasmids were transiently transfected into cells using Lipofectamine 2000 (Life Technologies, USA) according to the manufacturer’s instructions, and the stably transfected cell lines were screened by puromycin (5 μg/mL) or hygromycin B (100 µg/mL). When needed, cells were treated with cycloheximide (CHX; 100 μg/mL, Selleck, China) for 6 h before harvesting and then subjected to other analyses.

### 2.2 Plasmids construction

The short hairpin RNA (shRNAs) targeting AMBRA1 or CCND1 were synthesized as oligos (Biosune, Shanghai, China), annealed, and inserted into the pLKO.1 vector that was digested with EcoRI and AgeI (NEB, USA), the specific sequences for shRNAs as shown in Table 1. The single guide RNA (sgRNA) targeting AMBRA1 was designed using an online tool (http://crispr.mit.edu/), and the designed sgRNAs (Table 2) were synthesized as oligos (Biosune), annealed, and inserted into a PX330 vector that was digested with BbsI, generating PX330-AMBRA1-sgRNA. The plasmids containing AMBRA1 were amplified from the complementary DNA (cDNA) of human HEK293T cells and inserted into pCDH, pCADNA3.0-Flag, or pET28a vector. The plasmids of pET28a-His6-UB and pET28a-UBA1/UBCH5A/CUL4B/CCND1/USP2cc-His6 were kindly provided by Professor Weiwei Yang (Chinese Academy of Sciences, Shanghai, China).

**Table 1:** Sequences of the shRNAs targeting *AMBRA1*and *CCND1*

ShRNA	Target site sequence (5'-3')
Scramble	GCGCGATAGCGCTAATAATTT
shAMBRA1-1	GAGATTATCTCCTGCTGCATA
shAMBRA1-2	CTGATGAATGCCATTGGGCTT
shAMBRA1-3	GTGACCTGAGACGCTTCTTTC
shCCND1-1	CTCTAAGATGAAGGAGACCAT
shCCND1-2	GAACAAACAGATCATCCGCAA
shCCND1-3	CCACAGATGTGAAGTTCATTT

**Table 2:** Sequences of the sgRNA for *AMBRA1*

sgRNA	Target site sequence (5'-3')
sg AMBRA1	AAGTGTGTTCATTCCCTGAT

### 2.3 Reverse transcription quantitative PCR (RT-qPCR)

The total RNAs were extracted from cells using total RNA kit (Tiangen, Beijing, China). cDNA was synthesized using ReverTra Ace RT Master Mix (Toyobo, Osaka, Japan). qPCR assay was performed to assess the relative abundances of *AMBRA1* and *GAPDH* mRNAs using specific primers (Table 3), stained by SYBR Green (Tiangen) on the CFX96 real-time PCR system (Bio-Rad, California, USA). The relative abundances of *AMBRA1* were normalized to that of *GAPDH*, using the 2^ΔΔCt^ method [15]. All data were obtained from three independent experiments.

**Table 3:** Sequences of the primers used in RT-qPCR

Target gene	Forward primer (5'–3')	Reverse primer (5'–3')
GAPDH	GAGTCAACGGATTTGGTCGTATTG	ATTTGCCATGGGTGGAATCATATTG
AMBRA1	GTGGATGAAATGGGAGGGCAAGAG	CACTTGCCAGTCTTCACCTCCG

### 2.4 AMBRA1 knockout cell line

AMBRA1 knockout cell line was generated using the CRISPR-CAS9 (clustered regularly interspaced short palindromic repeats and CRISPR-associated protein 9) technique as previously described [16]. Briefly, the 92.1 cells were transfected with CRISPR-CAS9-based sgRNA (PX330-AMBRA1-sgRNA), and monoclonal were chosen and detected by immunoblotting analysis. Then, the genetic ablation of AMBRA1 was confirmed by Sanger sequencing.

### 2.5 Cell viability assay

Cells stably transfected with shRNAs targeting AMBRA1 or CCND1 or ectopically expressing PCDH-AMBRA1 were seeded into 96-well plates. Cell viability was determined using Cell Counting Kit-8 (CCK-8, Jiangsu, China) solution according to the manufacturer’s instructions at different time points, and the product was quantified spectrophotometrically at a wavelength of 450 nm using a microplate reader (Bio-Rad, USA). The 0 h time point was defined as 6 h after the cells were seeded. All experiments were conducted with six replicates and repeated three times.

### 2.6 Colony formation assay

One thousand cells stably transfected with PCDH or PCDH-AMBRA1 were seeded into six-well plates. Seven days later, plates were fixed with 4% paraformaldehyde (Merck, Darmstadt, Germany) at room temperature (RT) for 15 min and stained with 0.1% crystal violet (Beyotime, Jiangsu, China) for 30 min. Images were obtained by a camera (Sony, Tokyo, Japan), and the number of colonies was counted and calculated.

### 2.7 Co-immunoprecipitation (Co-IP), immunoprecipitation, and immunoblotting

For Co-IP, cells were lysed in 500 μL Co-IP buffer (50 mM Tris-HCl, 150 mM NaCl, 5 mM ethylenediaminetetraacetic acid (EDTA), and 1% Nonidet P-40 (NP-40), pH 7.5) supplemented with a protease inhibitor cocktail (Selleck, China) as previously described [9,17]. Subsequently, the cell lysates were centrifuged and incubated with anti-CCND1 antibody (1:100 dilutions; ab134175, Abcam, UK) and Protein G agarose beads (L-1006, Biolinkedin Biotech, China). For immunoprecipitation, cells were lysed in 800 μL immunoprecipitation buffer (50 mM Tris-HCl, 150 mM NaCl, 5 mM EDTA, 0.1% sodium dodecyl sulfate (SDS), and 1% NP-40, pH 7.4) supplemented with a protease inhibitor cocktail. Subsequently, the cell lysates were centrifuged and incubated with anti-CCND1 antibody and Protein G agarose beads, overnight at 4°C. The immunoprecipitates were enriched and denatured at 100°C for 10 min in 2× SDS-PAGE loading buffer. The inputs, immunoprecipitates, and other cell lysates were subjected to 10% SDS-PAGE (sodium dodecyl sulphate-polyacrylamide gel electrophoresis) and transferred to a polyvinylidene fluoride membrane (Bio-Rad). The membranes were blocked with 5% skimmed milk at RT for 1 h and immunoblotted with the specified antibodies as follows: anti-CCND1 (1:1,000 dilution, Abcam), anti-AMBRA1 (1:8,000 dilution; 13762-1-AP, Proteintech, USA), anti-ubiquitin (1:400 dilution; sc-47721, Santa Cruz, USA), and anti-GAPDH (1:10,000 dilution; 60004-1-Ig, Proteintech). Then the membranes were incubated with horseradish peroxidase conjugated goat antimouse IgG (1:5,000 dilution; SA00001-1, Proteintech) or goat antirabbit IgG (1:5,000 dilution; SA00001-2, Proteintech) secondary antibodies, and the signals were visualized using a Tanon 5200 Imaging System (Tanon, China).

### 2.8 Expression and purification of recombinant proteins

Recombinant proteins were purified as previously described [18]. His6-tagged proteins were expressed in BL21 *Escherichia coli* cells. After isopropyl-β-d-thiogalactopyranoside (Sangon) induction, the cells were pelleted, lysed in PBS buffer, and incubated with Ni^2+^ TA beads (Sangon) to enrich the respective proteins, followed by elution with 500 mM imidazole (Sangon) dissolved in PBS buffer and then dialysed in PBS buffer supplemented with 20% glycerol before being aliquoted and preserved at −80°C.

### 2.9 *In vitro* ubiquitination assay

*In vitro* ubiquitination assays were performed as previously described [18]. Briefly, recombinant 100 ng His6-Ub, 100 ng UBA1-His6 (E1), 100 ng UBCH5A-His6 (E2), 200 ng CUL4B(E3), 200 ng AMBRA1-His6, and 200 ng CCND1-His6 were added to ubiquitination buffer (25 mM Tris-Cl, 100 mM NaCl, 1 mM dithiothreitol, 5 mM MgCl_2_, 2 mM ATP, pH 7.4) with a final reaction volume of 25 μL and incubated at 37°C for 1.5 h. The ubiquitination levels of proteins were examined by immunoblotting assay using an anti-CCND1 antibody.

### 2.10 Statistical analysis

The data were analyzed by one-way ANOVA with Tukey’s *post hoc* test using GraphPad Prism 5 (GraphPad Software Inc., San Diego, USA). *P < 0.05 was considered to be significantly different; **P < 0.01 was considered to be very significantly different.

## 3 Results

### 3.1 AMBRA1 is lowly expressed in human UVM cells

**Figure 1:**
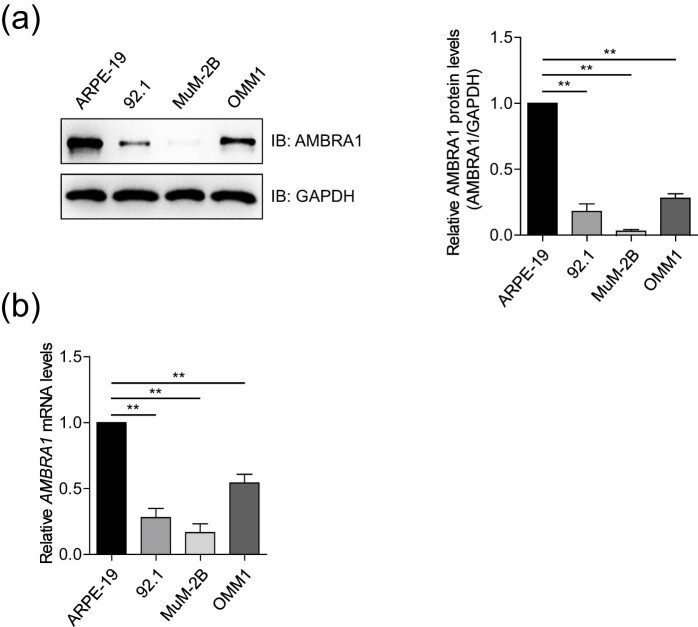
Expression profile of AMBRA1 in human pigment epithelial cells and UVM cells. (a) The protein levels of AMBRA1 in human pigment epithelial cells (ARPE-19) and UVM cell lines (92.1, MuM-2B, and OMM1) were detected by immunoblotting analysis. The bands of AMBRA1 were quantified and compared to that of GAPDH. The data are expressed as mean ± standard deviation (SD) and were analyzed using one-way ANOVA with Tukey’s *post hoc* test. **P < 0.01, very significantly different, and the experiment was repeated thrice. (b) The mRNA levels of *AMBRA1* in human pigment epithelial cells and UVM cells were detected by RT-qPCR. Data are expressed as the mean ± SD and were analyzed using one-way ANOVA with Tukey’s *post hoc* test. **P < 0.01, very significantly different, three independent experiments.

**Figure A1:**
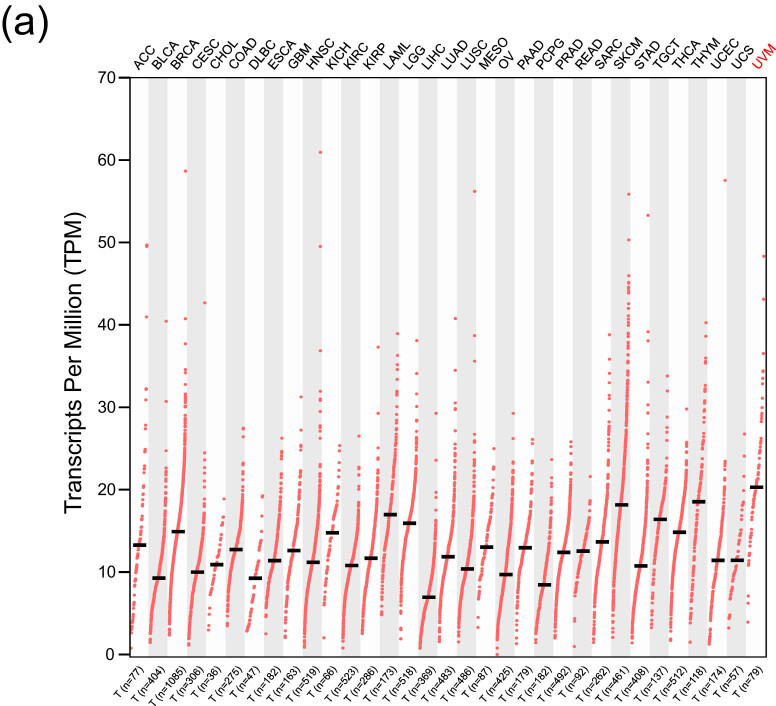
Expression of AMBRA1 across TCGA tumors, TCGA data was mined using the GEPIA2 (http://gepia.cancer-pku.cn/). UVM, Uveal Melanoma; T, Tumor tissue.

To explore the expression profile of AMBRA1 in human UM cell lines, the lysates of three human melanoma cell lines (92.1, MuM-2B, and OMM1) and one human pigment epithelial cell line (ARPE-19) were prepared. Immunoblotting analysis indicated that the protein levels of AMBRA1 were significantly higher in ARPE-19 cells compared to that of UVM cells (Figure 1a), which was consistent with the mRNA levels of *AMBRA1* detected by RT-qPCR (Figure 1b). However, AMBRA1 is highly expressed in UVM across tumors as revealed by TCGA (The Cancer Genome Atlas) data mined using the GEPIA2 (http://gepia.cancer-pku.cn/) (Figure A1a).

### 3.2 The ablation of AMBRA1 promotes the proliferation of UVM cells

**Figure 2:**
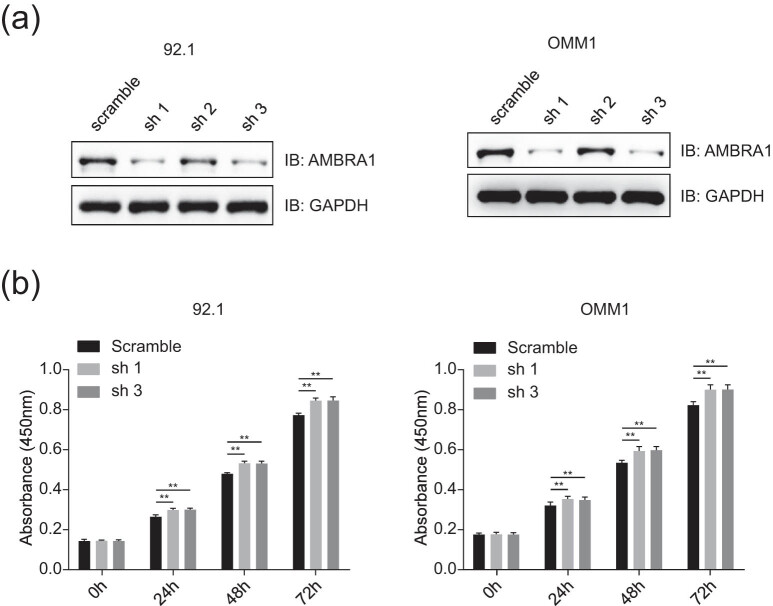
The ablation of AMBRA1 promotes the proliferation of UVM cells. (a) Test of the knockdown efficiency of shRNAs targeting AMBRA1 by immunoblotting analysis. The shRNAs for AMBRA1 were transfected into 92.1 and OMM1 cells, and then puromycin (5 µg/mL) selection was used to establish stably expressing cell lines before subjecting to immunoblotting analysis. (b) AMBRA1 knockdown promotes the proliferation of UVM cells. OMM1 and 92.1 cells stably expressing AMBRA1 shRNAs (sh 1 and sh 3) were seeded into 96-well plates, and the cell viability was detected using the CCK-8 assay at the indicated time points. The data were expressed as mean ± SD and analyzed using a one-way ANOVA with Tukey’s *post hoc* test. **P < 0.01, very significantly different, three independent experiments.

To explore the functions of AMBRA1 in UVM, three shRNAs targeting *AMBRA1* were designed and tested in 92.1 and OMM1 cells. Immunoblotting analysis indicated that AMBRA1 significantly decreased in cells stably transfected with shRNA1 and shRNA3 (Figure 2a), and these two shRNAs were chosen for further study. The cell viability of 92.1 and OMM1 was detected by the CCK-8 assay, and a significant cell growth promotion was observed in AMBRA1 knockdown cell lines compared to scramble groups (Figure 2b).

**Figure A2:**
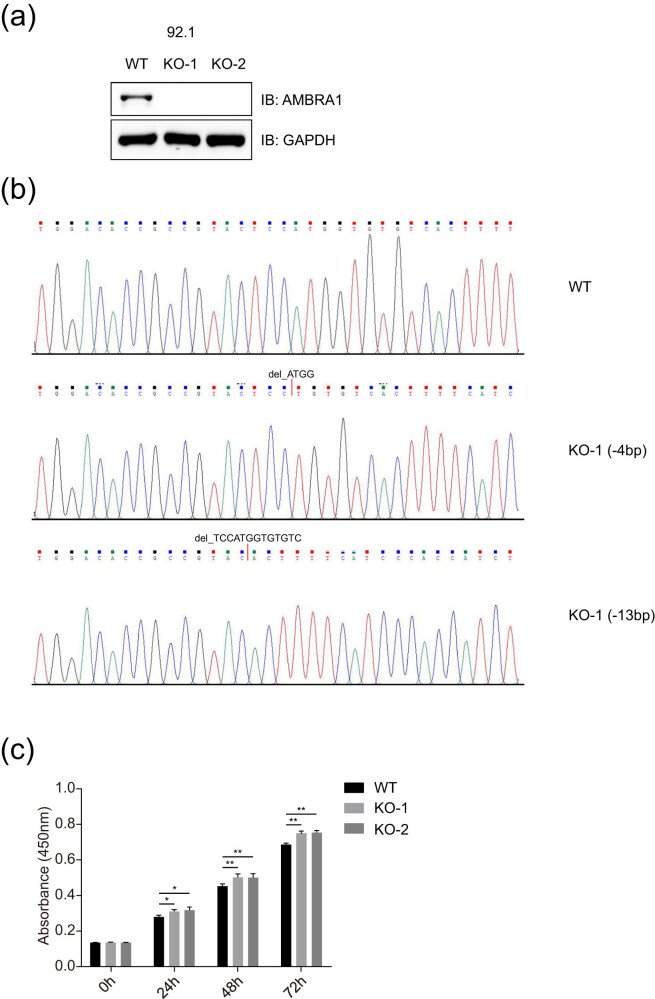
The knockout of AMBRA1 promotes the proliferation of 92.1 cells. (a and b) Two AMBRA1 knockout (KO) cell lines were generated using the CRISPR-CAS9 technique. 92.1 cells were transfected with CRISPR-CAS9-based sgRNA, monoclonal were picked and detected by immunoblotting analysis (a), and then the genetic ablation of AMBRA1 with a 4-bp and a 13-bp deletion were confirmed by Sanger sequencing (b). (c) AMBRA1 knockout promoted the proliferation of 92.1 cells. Wild type (WT) and AMBRA1 knockout 92.1 cells were seeded into 96-well plates, and the cell viability was detected using the CCK-8 assay at the indicated time points. The data were expressed as mean ± SD and analyzed using one-way ANOVA with Tukey’s post-hoc test. NS, no significantly different; **P < 0.01, very significantly different, the experiment was repeated three times.

Two AMBRA1 knockout 92.1 cell lines (KO-1 and KO-2) were generated using the CRISPR-CAS9 sgRNA-based method and validated by immunoblotting analysis and Sanger sequencing (Figure A2a and b). The viability of 92.1 cells was increased in AMBRA1 knockout cells compared to wild-type (WT) cells as revealed by the CCK-8 assay (Figure A2c).

### 3.3 AMBRA1 inhibits the proliferation of UVM cells

**Figure 3:**
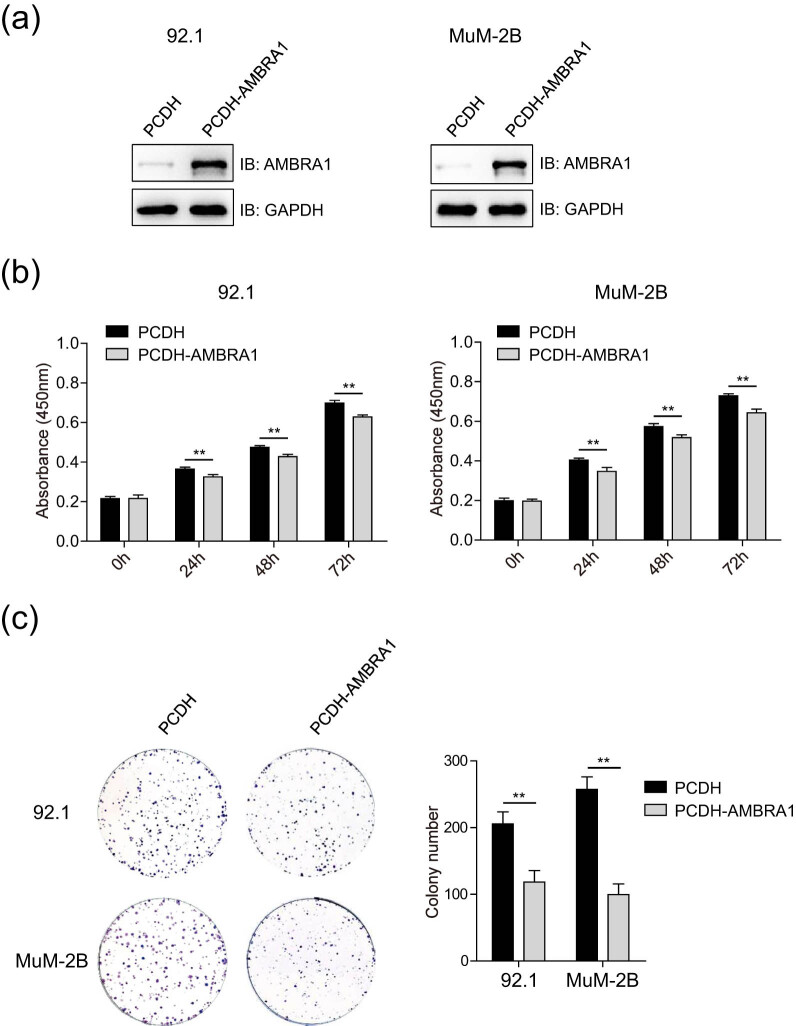
AMBRA1 inhibits the proliferation of UVM cells. (a) The protein levels of AMBRA1 in UVM cells stably expressing AMBRA1 were detected by immunoblotting analysis. MUM-2B and 92.1 cells were transfected with empty vector PCDH or PCDH-AMBRA1, and then puromycin (5 µg/mL) selection was used to establish stably expressing cell lines. (b) AMBRA1 inhibits the proliferation of UVM cells. MUM-2B and 92.1 cells stably expressing PCDH empty vector or PCDH-AMBRA1 were seeded into 96-well plates, and the cell viability was detected by the CCK-8 assay at the indicated time points. **P < 0.01, very significantly different, three independent experiments. (c) AMBRA1 inhibits the colony formation of UVM cells. MUM-2B and 92.1 cells stably expressing empty vector PCDH or PCDH-AMBRA1 were seeded into six-well plates, and the colonies were fixed and stained. The images were captured using a camera, and the colony numbers were counted and calculated. The data were expressed as mean ± SD and analyzed using one-way ANOVA with Tukey’s *post hoc* test. **P < 0.01, very significantly different, three samples for each group.

MuM-2B and 92.1 cells were stably transfected with empty vector PCDH or PCDH-AMBRA1 (Figure 3a). CCK-8 assay indicated that the cell viability of 92.1 and MuM-2B cells that stably expressing AMBRA1 was reduced than that of control groups (Figure 3b). Decreased colony numbers were observed in AMBRA1 overexpression groups compared to that of control groups, both in 92.1 and MuM-2B cell lines (Figure 3c). These results suggest that AMBRA1 inhibits the growth of UVM cells.

### 3.4 AMBRA1 reduces the stability of CCND1 protein

**Figure 4:**
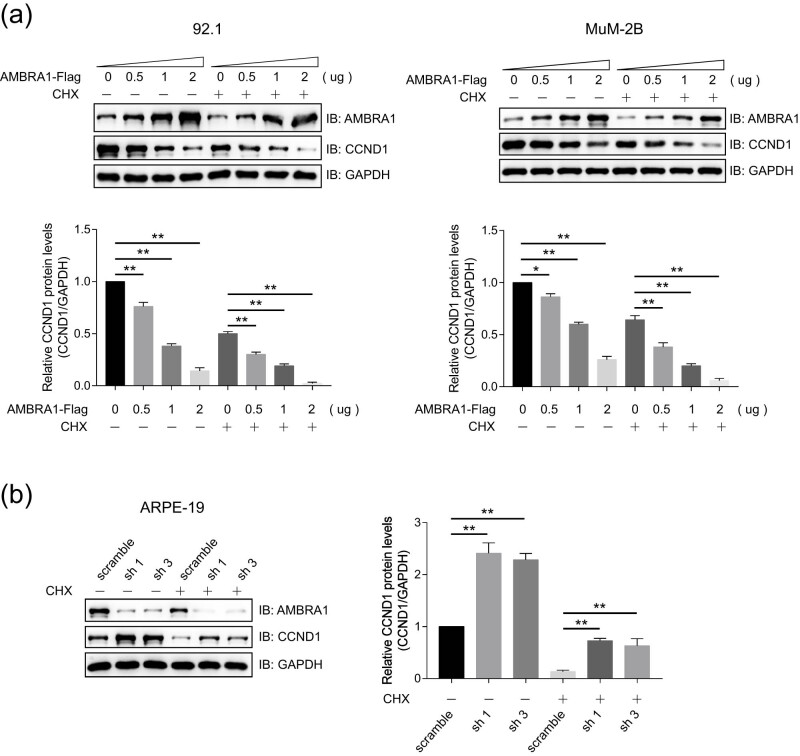
AMBRA1 reduces the stability of CCND1 protein. (a) AMBRA1 promoted the degradation of CCND1 in a dose-dependent manner. MUM-2B and 92.1 cells that were transiently transfected with different amounts of pCDNA3.0-AMBRA1-Flag or empty vectors, treated with or without 100 μg/mL CHX for 6 h, and then were subjected to immunoblotting analysis using indicated antibodies. The bands of CCND1 were quantified and compared to that of GAPDH. The data are expressed as mean ± SD and were analyzed using one-way ANOVA with Tukey’s *post hoc* test. **P < 0.01, very significantly different, and the experiment was repeated thrice. (b) The protein levels of CCND1 were upregulated in AMBRA1-knockdown cells. The protein levels of AMBRA1 and CCND1 were detected by immunoblotting analysis in ARPE-19 cells that stably express AMBRA1 shRNAs after treatment with or without CHX (100 μg/mL) for 6 h. The bands of CCND1 were quantified and compared to that of GAPDH. The data are expressed as mean ± SD and were analyzed using one-way ANOVA with Tukey’s *post hoc* test. **P < 0.05, significantly different; **P < 0.01, very significantly different, the experiment was repeated thrice.

**Figure A3:**
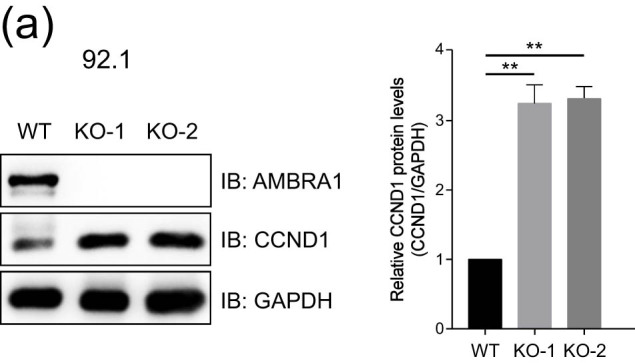
(a) The protein levels of CCND1 were upregulated in AMBRA1 knockout cells. The protein levels of AMBRA1 and CCND1 were detected by immunoblotting analysis in wild type and AMBRA1 knockout 92.1 cells. The bands of CCND1 were quantified and compared to that of GAPDH. The data are expressed as mean ± SD and were analyzed using one-way ANOVA with Tukey’s post-hoc test. **P < 0.01, very significantly different, the experiment was repeated three times.

The E3 ligase adaptor, AMBRA1, has been reported to regulate the stability of CCND1 [13,14], but its function in UVM has not been reported. Immunoblotting analysis indicated that AMBRA1 promoted the degradation of CCND1 in a dose-dependent manner both in 92.1 and MUM-2B cells (Figure 4a), and the protein levels of CCND1 were upregulated in AMBRA1 knockdown ARPE-19 cells (Figure 4b). Further study indicated that the protein levels of CCND1 were upregulated in AMBRA1 knockout 92.1 cells compared to that of WT cells (Figure A3a). These results suggested that AMBRA1 negatively regulates the stability of AMBRA1 protein.

### 3.5 AMBRA1 interacts with and promotes the ubiquitination of CCND1

**Figure 5:**
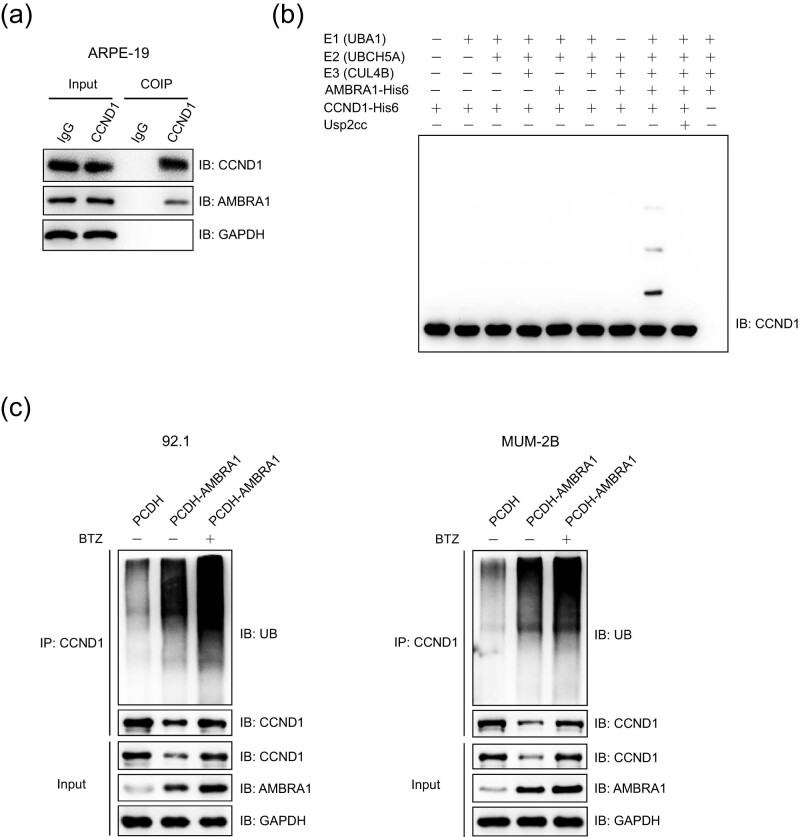
AMBRA1 interacts with and promotes the ubiquitination of CCND1. (a) Endogenous AMBRA1 interacted with CCND1. The lysates of ARPE-19 cells were immunoprecipitated with anti-IgG or anti-CCND1 antibody and detected by immunoblotting analysis. (b) AMBRA1 mediated the ubiquitination of CCND1 *in vitro*. An *in vitro* ubiquitination assay was carried out with the recombinant proteins E1 (UBA1-His6), E2 (UBCH5A-His6), E2 (CUL4B-His6), AMBRA1-His6, CCND1-His6, and His6-Ub, together with the indicated components, and subjected to immunoblotting analysis. Usp2cc, the catalytic core of human deubiquitinase Usp2. (c) AMBRA1 promoted the ubiquitination of endogenous CCND1. MUM-2B and 92.1 cells stably expressing empty vector PCDH or PCDH-AMBRA1 were treated with or without proteasome inhibitor BTZ (1 μM) for 6 h, and the lysates were immunoprecipitated with anti-CCND1 antibody before subjecting to immunoblotting analysis using indicated antibodies.

**Figure A4:**
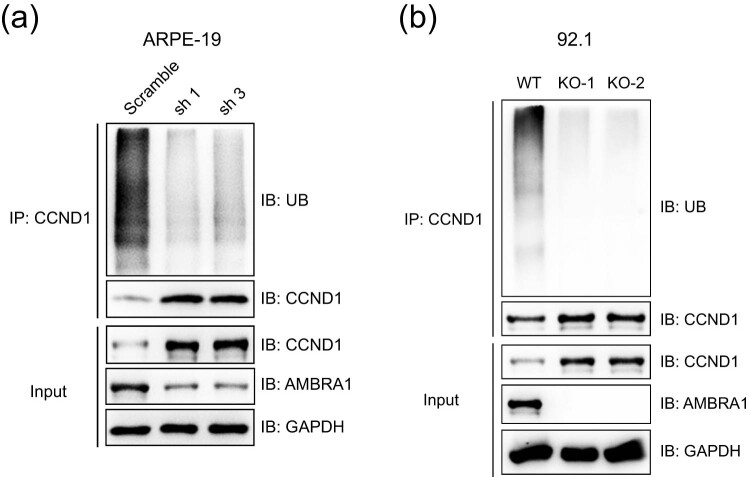
The ablation of AMBRA1 attenuates the ubiquitination of endogenous CCND1. (a) The knockdown of AMBRA1 attenuated the ubiquitination of endogenous CCND1. The lysates of ARPE-19 cells stably expressing AMBRA1 shRNAs were immunoprecipitated with anti-CCND1 antibody and subjected to immunoblotting analysis using indicated antibodies. (b) The knockout of AMBRA1 attenuated the ubiquitination of endogenous CCND1. The lysates of wild type and AMBRA1 knockout 92.1 cells were immunoprecipitated with anti-CCND1 antibody and subjected to immunoblotting analysis using indicated antibodies.

Co-IP analysis showed that endogenous AMBRA1 formed a complex with endogenous CCND1 in ARPE-19 cells (Figure 5a), and an *in vitro* ubiquitination assay was carried out with recombinant proteins indicated that AMBRA1 indeed mediates the ubiquitination of CCND1 (Figure 5b). As showed in Figure A4a and b, AMBRA1 knockdown or knockout significantly attenuated the ubiquitination of endogenous CCND1 in ARPE-19 cells or 92.1 cells. Further study indicated that the ubiquitination of CCND1 was significantly increased in 92.1 and MUM-2B cells that stably expressing PCDH-AMBRA1 compared to that of control groups, regardless of treated with proteasome inhibitor bortezomib (BTZ) or not (Figure 5c).

### 3.6 AMBRA1 regulates the proliferation of UVM cells in a CCND1-dependent manner

**Figure 6:**
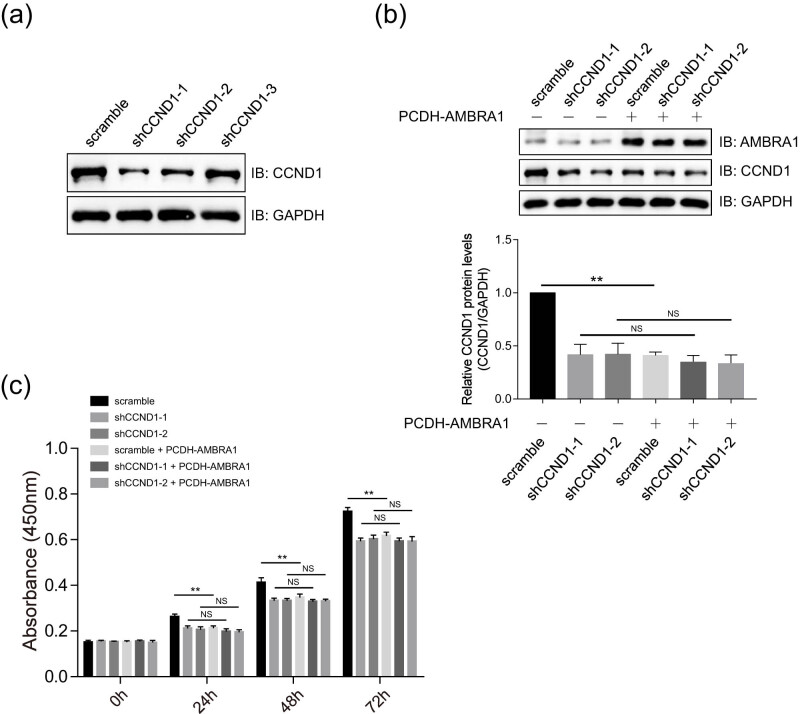
AMBRA1 regulates the proliferation of UVM cells in a CCND1-dependent manner. (a) Test of the knockdown efficiency of shRNAs targeting CCND1 by immunoblotting analysis. The shRNAs for CCND1 were transfected into 92.1 cells, and then hygromycin B (100 µg/mL) selection was used to establish stably expressing cell lines before subjecting to immunoblotting analysis. (b) Detection of the protein levels of AMBRA1 and CCND1 in stable expression cell lines. The 92.1 cells stably expressing CCND1 were transfected with an empty vector PCDH or PCDH-AMBRA1, selected by hygromycin B selection (20 µg/mL) and puromycin (5 µg/mL), and then subjected to immunoblotting analysis. The bands of CCND1 were quantified and compared to that of GAPDH. The data are expressed as mean ± SD and were analyzed using one-way ANOVA with Tukey’s *post hoc* test. NS, no significantly different; **P < 0.01, very significantly different, the experiment was repeated thrice. (c) AMBRA1 inhibited the proliferation of 92.1 cells in a CCND1-dependent manner. The 92.1 cells stably expressing shRNAs of CCND, and PCDH or PCDH-AMBRA1, were seeded into 96-well plates. The cell viability was detected using the CCK-8 assay at the indicated time points. The data were expressed as mean ± SD and were analyzed using one-way ANOVA with Tukey’s *post hoc* test. NS, no significantly different; **P < 0.01, very significantly different, and the experiment was repeated thrice.

Three shRNAs for CCND1 were designed and tested in 92.1 cells. Immunoblotting analysis indicated that CCND1 was significantly decreased in cells stably transfected with shCCND1-1 and shCCND1-2 (Figure 6a), and these two shRNAs were chosen for further study. As shown in Figure 6b, AMBRA1 showed little effect on the stability of CCND1 protein in CCND1-knockdown 92.1 cells compared to that of the scramble group (Figure 6b). AMBRA1 inhibited the proliferation of control group cells, but not CCND1-knockdown 92.1 cells, as revealed by the CCK-8 assay at different time points (Figure 6c). These data suggested that CCND1 is essential for AMBRA1-mediated growth inhibition.

## 4 Discussion

Recently, researchers from three collaborating teams of scientists in the United States and Europe found that AMBRA1 marks CCNDs involved in helping cells divide for destruction when cell division is not needed [13,14,19]. When the gene that produces AMBRA1 is damaged and the protein is missing or does not work properly, the cell cycle loses one of its main brakes, potentially letting cell division spiral out of control [13]. The loss of the AMBRA1 protein drives the tumor formation in mice and is linked with worse outcomes in some human tumors, and they also found that a lack of AMBRA1 may make some tumors resistant to drugs called CDK4/6 inhibitors, which are a promising new class of cancer treatments [14]. Thus, AMBRA1 serves as an important tumor suppressor by limiting tumor growth.

In this study, first, we found that AMBRA1 is lowly expressed in UM cells (Figure 1), whereas AMBRA1 is highly expressed in UVM across TCGA tumors (Figure A1a). For lack matched TCGA normal data, it is difficult to judge whether AMBRA1 is highly expressed in human pigment epithelial tissues compared to UVM tissues, which needs to be further explored. Then, we found that the ablation of AMBRA1 promoted the proliferation of 92.1 and OMM1 cells (Figure 2), which was somehow consistent with recently reported that loss of AMBRA1 promotes melanoma growth and invasion [20]. Next, we verified that ectopically expressing AMBRA1 inhibits the proliferation and colony formation of UVM cells (Figure 3). Other studies also reported that AMBRA1 controls antigen-driven activation and proliferation of naive T cells [21].

Cyclin D1 (CCND1) is an important regulator of G1 to S phase progression in many different cell types, which is important for the development and progression of several cancers, including those of the breast, esophagus, bladder, lung, and eye. [22,23]. The overexpression of CCND1 is a common event in cancer but does not occur solely as a consequence of gene amplification. Rather, increased levels of CCND1 frequently result from its defective regulation at the posttranslational level. Besides CRL4-AMBRA1 E3 ligase complex, other E3 ligases, for example, SKP2, FBXO31, HACE1, and FBXO4, were also reported that mediated the ubiquitination and degradation CCND1 [24,25,26,27]. AMBRA1 promotes the degradation of CCND1 both in 92.1 and MUM-2B cells, and AMBRA1 knockdown increases the protein levels of CCND1 in ARPE-19 cells (Figure 4a and b). These suggest that AMBRA1 acts as an important factor for CCND1 degradation in UVM. One weak point of our study is that no *in vivo* experiments were involved, and an ongoing effort is to test the function of AMBRA1-CCND1 axis in animal models and patient samples of UVM.
